# Prevalence and outcome of acute gastrointestinal injury in critically ill patients: A systematic review and meta-analysis

**DOI:** 10.1097/MD.0000000000012970

**Published:** 2018-10-26

**Authors:** Dong Zhang, Yuting Li, Lili Ding, Yao Fu, Xuechao Dong, Hongxiang Li

**Affiliations:** ICU, First Hospital of Jilin University, Changchun, China.

**Keywords:** acute gastrointestinal injury, classification, mortality, prevalence

## Abstract

**Background:**

The aim of the study was to investigate the prevalence and impact of acute gastrointestinal injury (AGI) on clinical outcomes in critically ill patients.

**Methods:**

The PubMed, Cochrane, and Embase databases were searched to identify trials that assessed gastrointestinal injury in critically ill patients. Outcome measures were prevalence of AGI among critically ill patients; incidence of mortality among critically ill patients with AGI, and incidence of mortality stratified by severity of AGI.

**Results:**

The meta-analysis included 14 studies. The prevalence of AGI in critically ill patients was 40% [95% confidence interval (CI), 27%–54%]; the incidence of mortality among critically ill patients with AGI was 33% (95% CI, 26%–41%). There was a higher risk of mortality in critically ill patients with AGI compared to those without AGI [risk ratio (RR) = 2.01; 95% CI 1.20–3.37, *P* = .008). Subgroup analyses of studies that defined AGI according to European Society of Intensive Care Medicine (ESICM) criteria confirmed these findings and showed that the risk of mortality was higher in critically ill patients with more severe AGI (ESICM grade III and IV vs grade II) [RR of 1.86 (95% CI 1.48–2.34), *P* < .00001].

**Conclusion:**

AGI is common in critically ill patients, mortality in critically ill patients with AGI is high, and severity of AGI is associated with mortality. The widespread clinical use of standard criteria with a severity gradation will facilitate the diagnosis and management of AGI in critically ill patients.

## Abstract 2

Supplemental Digital Content is available in the text

## 1 Introduction

The gastrointestinal (GI) tract is a complex organ system that performs digestive, absorptive, excretory, immune, endocrine, and barrier functions. GI symptoms occur in approximately 62% of patients in the intensive care unit (ICU), and may influence clinical outcomes.^[1]^

Traditionally, GI dysfunction or acute gastrointestinal injury (AGI) was evaluated based solely on the presence or absence of any specific GI symptom. In 2012, the Working Group on Abdominal Problems (WGAP) of the European Society of Intensive Care Medicine (ESICM) proposed a definition of AGI in intensive care patients as malfunctioning of the GI tract in critically ill patients due to their acute illness. Four grades of severity were identified: AGI grade I, a self-limiting condition with future risk of GI dysfunction or failure; AGI grade II (GI dysfunction), interventions are required to restore GI function; AGI grade III (GI failure), interventions cannot restore GI function; AGI grade IV, GI failure that is immediately life threatening.^[1]^

Although some studies have found the AGI grading scale is applicable for identifying the severity of GI dysfunction and could be used as a predictor of poor prognosis in patients in the ICU,^[2,3]^ others suggest that AGI has no influence on mortality in this patient population.^[4]^ The objective of this meta-analysis was to investigate the impact of AGI, using past definitions and that proposed by the ESICM WGAP, on clinical outcomes in critically ill patients.

## 2 Materials and methods

This systematic review and meta-analysis is reported according to the Preferred Reporting Items for Systematic Reviews and Meta-Analyses (PRISMA) guidelines.^[5]^ Ethical approval was not necessary for this study because it was a review of the published literature.

### 2.1 Search strategy

Two review authors independently searched the PubMed, Cochrane, and Embase databases from inception to the 31st of July 2017 using the following search terms: “feeding intolerance,” “food intolerance,” “feed intolerance,” “enteral tolerance,” “gastric tolerance,” “gastrointestinal tolerance,” “gastrointestinal symptoms,” “gastrointestinal injury,” “gastrointestinal dysfunction,” “critical care,” “critical illness,” “intensive care,” “intensive care unit,” “ICU,” and “critically ill.” A manual search of the reference lists from relevant articles was also carried out. The search was limited to publications in the English language. The search strategy for each database is summarized in Supplement 1.

### 2.2 Inclusion and exclusion criteria

Inclusion criteria were study design: prospective or retrospective observational cohort studies; population: any critically ill patients with AGI identified according to clearly defined criteria; intervention: consistent protocol of enteral feeding across patients with or without GI dysfunction; outcomes: mortality.

Exclusion criteria were reviews, letters, abstracts, or editorials; studies that reported insufficient data; and studies that only included patients with burns or malignancy.

In this study, AGI and its 4 grades of severity were defined according to the recommendations of the ESICM WGAP, except AGI grade I, which was considered non-AGI.^[1]^

### 2.3 Data extraction

Two review authors independently examined titles and abstracts to select eligible studies. The full text of potentially relevant studies was retrieved and examined to determine which studies met the inclusion criteria. Disagreements about the study selection were resolved by discussion and consensus.

Two review authors independently extracted data from eligible studies, including study design and setting, study inclusion criteria, definition of GI dysfunction, AGI classification (if available), and incidence of mortality in the ICU. Disagreements about data extraction were resolved by discussion and consensus.

### 2.4 Quality assessment

Two review authors independently assessed the methodological quality of the included studies using the Newcastle-Ottawa scale (NOS),^[6]^ which allocates a maximum of 9 points according to the quality of the selection, comparability, and outcomes of the study populations. Study quality was defined as poor (0–3), fair (4–6), or good (7–9). Publication bias was not assessed, because each pooled estimate included <10 studies.

Disagreements about assessment of methodological quality were resolved by discussion and consensus.

### 2.5 Statistical analysis

Statistical analyses were performed using Review Manager Version 5.3 (RevMan, Cochrane Collaboration). For incidence outcomes, the reported incidence and standard deviation for AGI were calculated, and inverse variance was used to represent incidence as a risk difference. Risk ratios (RRs) with 95% confidence intervals (CIs) were calculated for dichotomous variables. A random-effects model was used to pool studies with significant heterogeneity, as determined by the chi-squared test (*P* ≤ .10) and inconsistency index (*I*^*2*^ ≥ 50%).^[7]^ Subgroup analyses were conducted using the subset of studies that defined AGI according to ESICM WGAP criteria. A sensitivity analysis was performed, omitting 1 study at a time, to investigate the effect of each study on the association between AGI and mortality. *P* < .05 was considered statistically significant.

## 3 Results

### 3.1 Study characteristics and quality assessment

**Figure 1:**
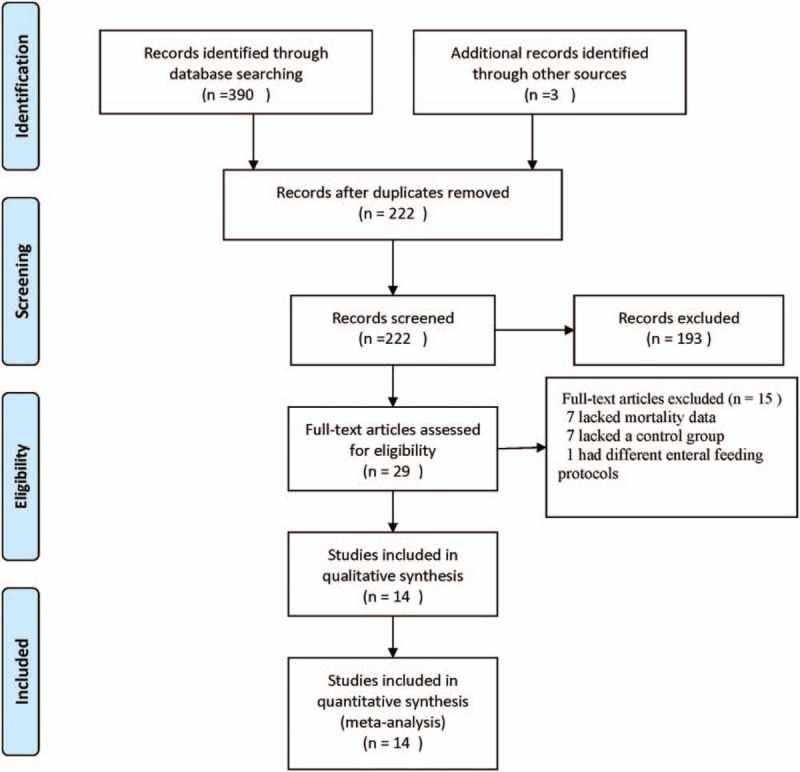
Flow chart of the study selection.

The search identified 393 articles. The titles and abstracts were screened, and 29 studies were considered potentially eligible for inclusion. Full-text articles were retrieved. After analyzing these full-text articles, 15 studies were excluded. Among these studies, 7 studies lacked mortality data, 7 studies lacked a control group, and 1 study used different enteral feeding protocols for patients with AGI and controls. Finally, 14 studies^[2–4,8–18]^ were found to be eligible for inclusion in our review (Fig. 1).

The characteristics of the included studies are shown in Table 1. A total of 14 eligible studies were published between 1987 and 2017. Among these studies, 4 studies were conducted in China, 6 studies were conducted in Europe, 1 study was conducted in Japan, 1 study was conducted in Saudi Arabia, 1 study was conducted in Canada, and 1 study involved 21 countries. A variety of criteria were used to define AGI. Among the included studies, only 4 studies applied the ESICM WGAP criteria. Overall, these studies included 8565 patients; of these, 2977 patients experienced AGI. The methodological quality of the included studies was good, and the mean NOS score was 8.5 (Table 2).

**Table 1:**
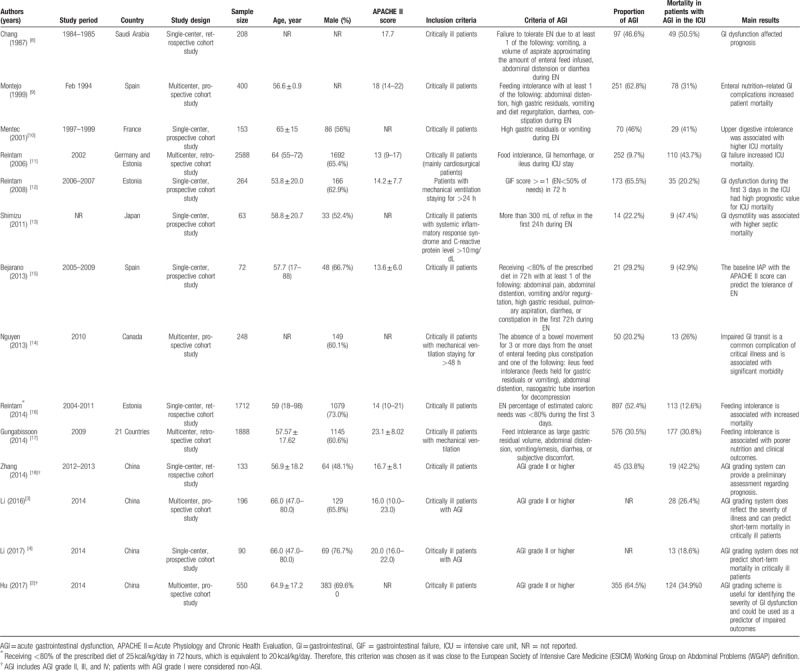
Characteristics of the included studies.

**Table 2:**
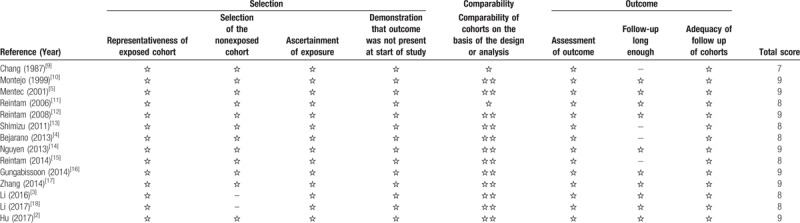
Quality of the included studies (Newcastle-Ottawa quality assessment scale).

### 3.2 Prevalence of AGI

**Figure 2:**
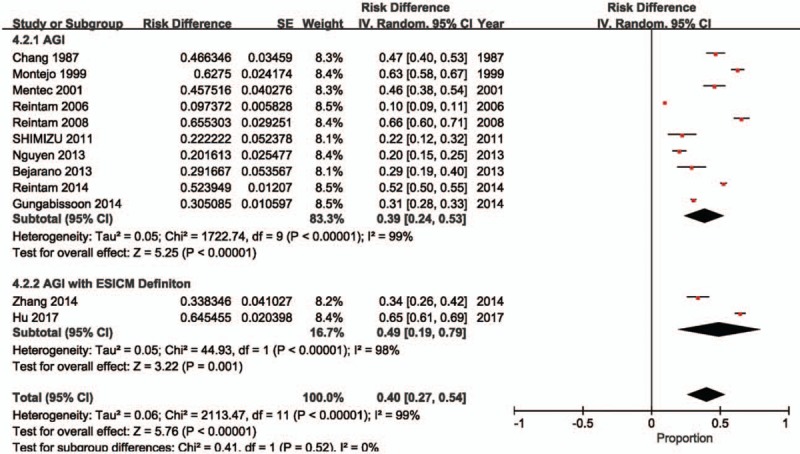
Incidence of AGI in critically ill patients. AGI = acute gastrointestinal injury, CI = confidence interval, ESICM = European Society of Intensive Care Medicine.

Two studies^[2–4,18]^ did not include a control group; therefore, the prevalence of AGI in critically ill patients is reported in 12 studies^[2,8–18]^ (n = 8279 patients; of these, 2081 patients experienced AGI). The meta-analysis estimated the prevalence of AGI in these critically ill patients at 40% (95% CI: 27%–54%). There was evidence of substantial heterogeneity between studies (*P* < .00001, *I*^2^ = 99%; Fig. 2).

### 3.3 Mortality associated with AGI

**Figure 3:**
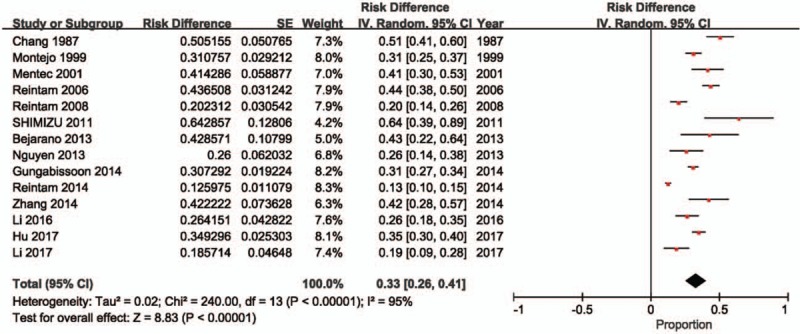
Incidence of mortality in patients with acute gastrointestinal injury (AGI). CI = confidence interval.

The incidence of mortality in critically ill patients with AGI is reported in 14 studies^[2–4,8–18]^ (n = 2977 patients with AGI). The meta-analysis estimated the incidence of mortality among these critically ill patients with AGI at 33% (95% CI: 26%–41%). There was evidence of substantial heterogeneity between studies (*P* < .00001, *I*^2^ = 95%; Fig. 3).

**Figure 4:**
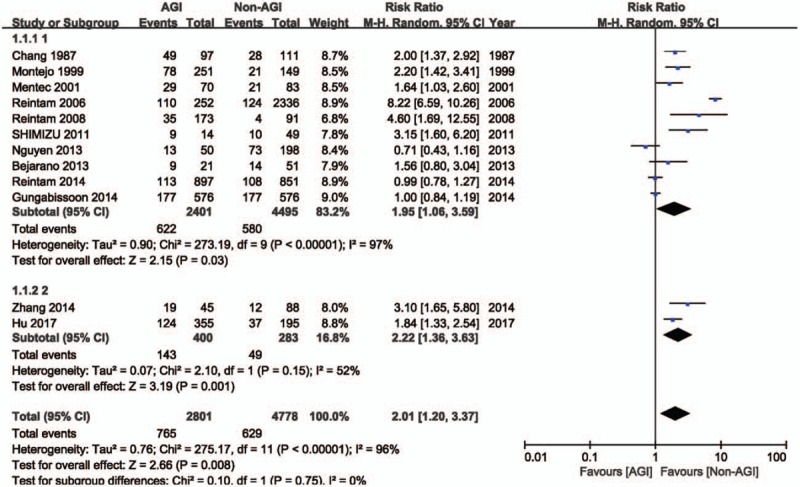
Risk of mortality in patients with AGI. AGI = acute gastrointestinal injury, CI = confidence interval.

Risk of mortality in critically ill patients with AGI is reported in 12 studies (n = 7579 patients; of these, 2801 patients experienced AGI).^[2,8–18]^ The meta-analysis demonstrated a higher risk of mortality in critically ill patients with AGI compared to those without AGI (RR: 2.01, 95% CI: 1.20–3.37, *P* = .008). There was evidence of substantial heterogeneity between studies (*P* < .00001, *I*^2^ = 96%; Fig. 4).

### 3.4 Subgroup analyses

Four studies^[2–4,18]^ that defined AGI according to ESICM WGAP criteria (n = 969 patients; of these, 576 patients experienced AGI) were included in the subgroup analysis.

Two of these studies^[2,18]^ reported the incidence of AGI and risk of mortality. The meta-analysis estimated the incidence of AGI defined according to ESICM WGAP criteria in critically ill patients at 49% (95% CI: 19%–79%; Fig. 2), and demonstrated that the risk of mortality was higher in critically ill patients with AGI defined according to ESICM WGAP criteria compared to those without AGI (RR: 2.22, 95% CI: 1.36–3.63, *P* = .001; Fig. 4). There was evidence of substantial heterogeneity between studies (incidence estimate: *P* < .00001, *I*^2^ = 98%; mortality estimate: *P* = .15, *I*^2^ = 52%).

**Figure 5:**
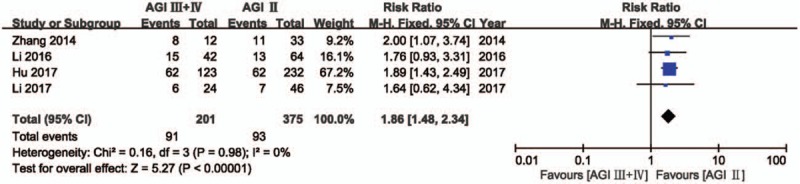
Risk of mortality according to the grade of AGI severity. AGI = acute gastrointestinal injury, CI = confidence interval.

All 4 studies reported the incidence of mortality stratified according to the grade of AGI severity (grade III and IV vs grade II), as defined by the ESICM WGAP (n = 576 patients with AGI; of these, 201 patients experienced AGI grade III and IV, and 375 patients experienced AGI grade II). The meta-analysis demonstrated a higher risk of mortality in critically ill patients with AGI grade III and IV, compared to those with AGI grade II (RR: 1.86, 95% CI: 1.48–2.34, *P* < .00001). There was no evidence of heterogeneity between studies (*P* = .98, *I*^2^ = 0%, Fig. 5).

### 3.5 Publication bias and sensitivity analysis

Publication bias was not assessed, because each pooled estimate included <10 studies.

Sensitivity analysis that omitted 1 study at a time produced RRs between 1.66 (1.25–2.21) and 2.21 (1.29–3.79), indicating that the results of this meta-analysis are robust.

## 4 Discussion

This meta-analysis was conducted to investigate the impact of AGI on clinical outcomes in critically ill patients. The findings revealed a higher risk of mortality in critically ill patients with AGI compared to those without AGI. In addition, mortality risk was increased in critically ill patients with more severe AGI, compared to patients with less severe AGI (ESICM WGAP grade III and IV vs grade II).

Most studies included in this meta-analysis reported a high prevalence of AGI in critically ill patients. However, the variation between estimates was high. As the prevalence of AGI depends heavily on the definitions applied, the disparity between studies likely resulted from the different criteria applied to identify AGI. In 2012, the ESICM WGAP recommended standardized criteria to define and grade the severity of AGI in critically ill patients. In the present review, the subgroup analysis of studies that applied the standardized ESICM WGAP criteria demonstrated that AGI occurs in approximately 50% of patients in critical care. In accordance with these findings, evidence suggests that almost 50% of patients admitted to the ICU have enterocyte damage.^[19]^

In critically ill patients, AGI may manifest as delayed gastric emptying, changes in intestinal motility patterns, and impaired integrity of the intestinal barrier.^[1]^ Dysfunction in these processes decreases nutrient absorption, leading to malnutrition.^[20]^ AGI may also result from or augment systemic inflammatory reaction syndrome and multiple organ dysfunction syndrome (MODS), in which the release of inflammatory mediators following trauma, surgery, infection, and hemorrhage causes intestinal flora translocation and injury to the intestinal mucus membrane, and results in loss of barrier function, an impaired immune-protective system, and secretion dysfunction.^[21,22]^ Some evidence suggests that the development of MODS is associated with a derangement in intestinal permeability, which is detectable before the onset of MODS,^[23]^ and that GI dysfunction serves as the main driver of MODS in injured or critically ill patients.^[12]^ Interestingly, incremental organ failure in MODS results in a 20% increase in mortality.^[24]^ Similarly, in the present study, the mortality of AGI patients was significantly higher than that of non-AGI patients, and mortality increased in patients with GI failure, compared to patients with GI dysfunction.

Assessment of GI function is difficult because some of the symptoms are subjective and poorly defined,^[25]^ which may be one reason why studies on AGI in critically ill patients cannot be standardized. One study showed that a combination of the GI failure score and the Sequential Organ Failure Assessment (SOFA) score had good prognostic value in patients who were mechanically ventilated on admission to hospital and stayed in the ICU for longer than 24 hours. Another study found that an increasing number of GI symptoms independently predicted 28-day mortality, but an additional dysfunction score that significantly improved the prognostic accuracy of the SOFA score could not be developed due to data set limitations, definition problems, or possibly because GI dysfunction was the secondary cause of other organ failure.^[25]^ In the present review, included studies defined GI dysfunction according to disparate criteria, which may explain the substantial heterogeneity in this meta-analysis. Conversely, there was no heterogeneity between the 4 studies that reported on the incidence of mortality stratified according to the grades of AGI severity identified by the ESICM WGAP. Because of the lack of markers for the measurement of GI function, the definition of AGI proposed by the ESICM WGAP is based on GI symptoms; therefore, establishing objective criteria for diagnosing AGI remains an urgent unmet need.

This review was associated with several limitations. First, the number of included studies was small. In the future, large, multicenter prospective observational studies are required to accurately characterize AGI and understand its impact on the morbidity and mortality of critically ill patients. Second, there was substantial heterogeneity among the included studies. Therefore, our findings should be interpreted with caution. This heterogeneity may have arisen from the disparate criteria used across the included studies to define AGI. The establishment of the ESICM WGAP criteria as a standard may facilitate the diagnosis of AGI in critically ill patients. Third, publication bias was not assessed due to the small sample size. Last, the primary disease and comorbidities of patients were not considered in the present analysis. However, all included studies recognized AGI as an independent pathophysiology.

## 5 Conclusions

This meta-analysis demonstrated that AGI is common in critically ill patients, mortality in critically ill patients with AGI is high, and severity of AGI is associated with mortality. The widespread clinical use of standard criteria with a severity gradation will facilitate the diagnosis and management of AGI in critically ill patients.

## Author contributions

**Conceptualization:** Hongxiang Li.

**Data curation:** Yao Fu.

**Investigation:** Lili Ding.

**Methodology:** Xuechao Dong.

**Software:** Yao Fu.

**Supervision:** Hongxiang Li.

**Validation:** Lili Ding.

**Visualization:** Dong Zhang.

**Writing – original draft:** Dong Zhang.

**Writing – review and editing:** Dong Zhang, Yuting Li, Hongxiang Li.

Hongxiang Li orcid: 0000-0002-1399-8039.

## Supplementary Material

Supplemental Digital Content
