# Supplementary material for: Prevalence and outcome of acute gastrointestinal injury in critically ill patients: A systematic review and meta-analysis
Source: Medicine (Baltimore). 2018 Oct 26;97(43):e12970. doi: 10.1097/MD.0000000000012970 (PMC6221717; doi:10.1097/MD.0000000000012970)
Supplement: Supplemental Digital Content [file medi-97-e12970-s001.doc]

**In pubmed:**

((((((((feeding intolerance) OR food intolerance) OR feed intolerance) OR enteral tolerance) OR gastric tolerance) OR gastrointestinal tolerance)) AND ((((((critical care[MeSH Terms]) OR critical care[Title/Abstract]) OR critical illness[Title/Abstract]) OR intensive care[Title/Abstract]) OR intensive care unit[Title/Abstract]) OR critically ill[Title/Abstract])) AND ((((((((((observation[MeSH Terms]) OR observational study[Title/Abstract]) OR observational studies[Title/Abstract]) OR observational study[Publication Type]) OR retrospective study[Title/Abstract]) OR retrospective studies[Title/Abstract]) OR retrospective study[Publication Type])) AND English[Language]) NOT review[Publication Type])

Result: 34 full-articles

(((((gastrointestinal symptoms) OR gastrointestinal injury) OR gastrointestinal dysfunction)) AND ((((((critical care[MeSH Terms]) OR critical care[Title/Abstract]) OR critical illness[Title/Abstract]) OR intensive care[Title/Abstract]) OR intensive care unit[Title/Abstract]) OR critically ill[Title/Abstract])) AND ((((((((((observation[MeSH Terms]) OR observational study[Title/Abstract]) OR observational studies[Title/Abstract]) OR observational study[Publication Type]) OR retrospective study[Title/Abstract]) OR retrospective studies[Title/Abstract]) OR retrospective study[Publication Type])) AND English[Language]) NOT review[Publication Type])

Result: 174 full-articles

**In Cochrane databases:**

(“feeding intolerance” OR “food intolerance” OR “feed intolerance” OR “enteral tolerance” OR “gastric tolerance” OR “gastrointestinal tolerance” OR “gastrointestinal symptoms” OR “gastrointestinal injury” OR “gastrointestinal dysfunction” ) AND (“critical care” OR “critical illness” OR “intensive care” “intensive care unit” OR “critically ill”)

Result: 92 full-articles

**In Embase**

(“feeding intolerance” OR “food intolerance” OR “feed intolerance” OR “enteral tolerance” OR “gastric tolerance” OR “gastrointestinal tolerance” OR “gastrointestinal symptoms” OR “gastrointestinal injury” OR “gastrointestinal dysfunction” ) AND (“critical care” OR “critical illness” OR “intensive care” “intensive care unit” OR “critically ill”)

Study types: Observational Study and Retrospective Study

Result: 93 full-articles
